# Structural mechanism of heavy metal-associated integrated domain engineering of paired nucleotide-binding and leucine-rich repeat proteins in rice

**DOI:** 10.3389/fpls.2023.1187372

**Published:** 2023-06-28

**Authors:** Liwei Guo, Yuanyu Mu, Dongli Wang, Chen Ye, Shusheng Zhu, Hong Cai, Youyong Zhu, Youliang Peng, Junfeng Liu, Xiahong He

**Affiliations:** ^1^ State Key Laboratory for Conservation and Utilization of Bio-Resources, Yunnan Agricultural University, Kunming, Yunnan, China; ^2^ Key Laboratory of Agro-Biodiversity and Pest Management of Education Ministry of China, Yunnan Agricultural University, Kunming, Yunnan, China; ^3^ State Key Laboratory of Agrobiotechnology, China Agricultural University, Beijing, China; ^4^ Key Laboratory of Forest Resources Conservation and Utilization in the Southwest Mountains of China Ministry of Education, Southwest Forestry University, Kunming, Yunnan, China

**Keywords:** protein engineering, molecular structure, paired NLRs, HMA IDs, rice

## Abstract

Plant nucleotide-binding and leucine-rich repeat (NLR) proteins are immune sensors that detect pathogen effectors and initiate a strong immune response. In many cases, single NLR proteins are sufficient for both effector recognition and signaling activation. These proteins possess a conserved architecture, including a C-terminal leucine-rich repeat (LRR) domain, a central nucleotide-binding (NB) domain, and a variable N-terminal domain. Nevertheless, many paired NLRs linked in a head-to-head configuration have now been identified. The ones carrying integrated domains (IDs) can recognize pathogen effector proteins by various modes; these are known as sensor NLR (sNLR) proteins. Structural and biochemical studies have provided insights into the molecular basis of heavy metal-associated IDs (HMA IDs) from paired NLRs in rice and revealed the co-evolution between pathogens and hosts by combining naturally occurring favorable interactions across diverse interfaces. Focusing on structural and molecular models, here we highlight advances in structure-guided engineering to expand and enhance the response profile of paired NLR-HMA IDs in rice to variants of the rice blast pathogen MAX-effectors (*Magnaporthe oryzae* AVRs and ToxB-like). These results demonstrate that the HMA IDs-based design of rice materials with broad and enhanced resistance profiles possesses great application potential but also face considerable challenges.

## 1 Introduction

Rice (*Oryza sativa*) is one of the three main cash crops worldwide and a staple food of more than half the world’s population. However, rice production is constantly threatened by diseases. *Magnaporthe oryzae* that causes rice blast, also known as “the cancer of rice,” was ranked first in the top 10 list of fungal plant pathogens, annually destroying enough rice to feed more than 200 million people for a year (Dean et al., 2012; Fisher et al., 2012; Savary et al., 2019). Therefore, identifying effective approaches to reduce rice yield loss is a clear ongoing need to secure rice supplies and meet the high global demand for food.

Plants differ from animals because they lack circulating immune cells for the interception of microbial signals. Therefore, during their interactions with microbes, plants depend on the cell-autonomous innate immune system of each individual cell to sense and respond to the signaling molecules of microbes. The activation of plant defense responses is triggered by the recognition of invading organisms by immune receptors. However, when pathogens evolve strateges to interfere with host defenses, the immune system is rendered nullified. The ensuing failure of the plant immune system allows further ingress of invading pathogens, resulting in disease susceptibility in plants (Lapin and Van den Ackerveken, 2013).

Molecular mechanisms of plant–pathogen interactions have demonstrated that most nucleotide-binding and leucine-rich repeat (NLR) domains are crucial downstream components of host resistance (Ausubel, 2005; Jones and Dangl, 2006; Jacob et al., 2013; Böhm et al., 2014; Zhang et al., 2022). Plants possess several *R* genes, most of which encode NLR receptors, and serve important functions, such as molecular exchange and signal transduction in the regulation of immune responses, biological stress, and apoptosis (Collier and Moffett, 2009; Jacob et al., 2013; Bernoux et al., 2016). However, the continuous evolution of virulent strains of pathogens hinders the management of gene-for-gene diseases such as rice blast caused by *M. oryzae*. Several strategies have been developed to consider resistance erosion, including genetic engineering of *R* genes. In recent years, many researchers have confirmed that several NLR proteins contain non-canonical integrated domains (IDs), which recognize avirulence (AVR) effectors and trigger immune responses. In particular, the heavy metal-associated (HMA) IDs in rice have been targeted as candidate-engineering elements for anti-rice blast. Therefore, a deep understanding of the molecular mechanisms of plant–pathogen interactions and further exploration and use of plant immunity for disease control are crucial to promote the sustainable development of green agriculture. In this review, we discuss the immune systems of plants and distinctive structures of NLR pairs. We also highlight the progress made in exploiting the structural basis of interactions between paired NLR-HMA IDs of rice and MAX-effectors (*M. oryzae* AVRs and ToxB-like) for *R* gene engineering. Finally, we discuss the prospects that need to be addressed to pave the way for the development of broad-race, spectrum-resistant cultivars.

## 2 Plant immune system

With the co-evolution of hosts and host-adapted microbes, plants have developed diverse immune strategies to combat microbial pathogens (Eitas and Dangl, 2010; Yang et al., 2013). In the prepenetration phase, the cell wall is the first natural physical and defensive barrier of plants and is known to exert basal immune responses. Once pathogens are recognized, cell wall-associated defense appears to operate *via* the inhibition of fungal cell wall-degrading enzymes, secretion of fungitoxic peptides and phytoalexins, and cell wall strengthening (Hückelhoven, 2007). When pathogens enter host cells, a crosstalk between classical pathogen-associated molecular pattern (PAMP)-triggered immunity (PTI) and effector-triggered immunity (ETI) is coordinated to initiate immune responses (Böhm et al., 2014; Zipfel, 2014). Signaling initiated by pattern recognition receptors (PRRs) and NLR proteins leads to overlapping downstream cellular responses, including defense gene expression, production of reactive oxygen species (ROS), and callose deposition, which may cause local cellular suicide and prevent further growth of biotrophic pathogens (Jones and Dangl, 2006; Tsuda and Katagiri, 2010).

On the cell surface, receptor-like kinases (RLKs) and receptor-like proteins (RLPs) function as PRRs that act in the first tier of the plant immune system to respond to PAMPs (Boller and Felix, 2009). The extracellular domains (ECDs) of PRRs are highly diverse, and PRRs can be subdivided according to the nature of their ligand-binding ectodomains (Boller and Felix, 2009; Couto and Zipfel, 2016; Sun et al., 2022). One large class of PRRs includes leucine-rich repeats (LRR) (Boller and Felix, 2009; Couto and Zipfel, 2016; Sun et al., 2022). LRRs are ECDs of RLKs and RLPs that bind chitin and peptidoglycan (Kaku et al., 2006; Williams et al., 2011; Willmann et al., 2011; Williams et al., 2014). The main difference between LRR-RLKs and LRR-RLPs is that the former comprises ECDs, transmembrane domain, and intracellular kinase domain (IKD), whereas the latter lacks an IKD (Ade et al., 2007; Monaghan and Zipfel, 2012). Other existing ECDs include lectin motifs and epidermal growth factor-like domains (Böhm et al., 2014). Generally, LRR-containing RLKs and RLPs act as sensors of peptide PAMPs (Monaghan and Zipfel, 2012). The initiated PPR signaling results in PTI responses, like stomatal closure, ion flow, ROS burst, gene expression changes, and aggregation of antimicrobial active substances (Göhre and Robatzek, 2008; Wirthmueller et al., 2013) that ward off microorganisms.

To establish successful infections, pathogens have evolved a diverse repertoire of effectors that are delivered to plant cells to interfere with PTI (Jones and Dangl, 2006). These effectors are characterized by low molecular weight (≤300 amino acids), presence of a signal peptide at the N-terminus, high cysteine content, and low sequence similarity (Donofrio and Raman, 2012). To counter effector infection, plants use specific NLR receptor proteins to detect pathogen-secreted effector proteins either through direct or indirect binding and activate high-level defense responses of the host (Hogenhout et al., 2009; Tsuda and Katagiri, 2010; Cesari et al., 2014a; Cesari et al., 2014b; Selin et al., 2016). Recognition of pathogen effectors results in effector-triggered immunity (ETI).

Although the two classes of immune receptors involve different activation mechanisms and appear to require different early signaling components, a crosstalk between PTI and ETI appears to be mediated by an integrated signaling network (Tsuda and Katagiri, 2010; Yuan et al., 2021a; Yuan et al., 2021b). ETI enhances, but does not initiate, PTI-induced defense responses by upregulating PTI signaling components. In turn, PTI potentiates ETI-induced cell death. Thus, immune pathways activated by the cell surface and intracellular receptors in plants mutually potentiate the activation of strong defenses against pathogens (Ngou et al., 2021; Yuan et al., 2021b).

## 3 Structural characteristics and functions of NLR protein pairs

In most cases, the recognition and interactions between plants and pathogens are believed to be consistent with the canonical gene-for-gene hypothesis (Flor, 1971). However, several recent studies have demonstrated that single *R* genes are inadequate for defense against pathogen invasion and necessitate complementary paired *R* genes encoding for defense responses (Bomblies et al., 2007; Ashikawa et al., 2008; Cesari et al., 2013; Cesari et al., 2014a; Cesari et al., 2014b). NLR protein pairs comprise two structurally and functionally different proteins encoded by paired *R* genes closely linked in a head-to-head configuration that can form homologous or heterologous complexes within the host (Eitas and Dangl, 2010; Kanzaki et al., 2012; Adachi et al., 2019). NLR proteins are multidomain proteins that possess a conserved architecture, including a C-terminal LRR domain, a central nucleotide-binding and oligomerization domain (NOD), and a variable N-terminal domain (Takken and Goverse, 2012). The N-terminal domains mainly include the Toll-interleukin 1 receptor (TIR)-like or coiled-coil (CC) types; thus, NLR proteins can be largely classified into TIR-NLR (TNL) and CC-NLR (CNL) based on differences in N-terminal structure (Pan et al., 2000; DeYoung and Innes, 2006). Many studies have established the CC and TIR domains as signaling modules (Lapin et al., 2019; Lapin et al., 2022; Zhang et al., 2022).

In the absence of pathogens, NLR paired proteins are maintained in an inactive state. Structure-function analyses have shown that the particular motifs of NLR play critical roles in their auto-inhibition mechanisms. For a number of NLR proteins, the “inactive” state has been shown to be associated with ADP binding (Wang et al., 2019a). The NOD of NLRs contains ADP/ATP-binding motif (P-loop) and is believed to function as a nucleotide switch (Lukasik and Takken, 2009; Wang and Chai, 2020). An ADP molecule binds to pocket formed by tight packing of the three subdomains, nucleotide binding domain (NBD), helical domain 1 (HD1), and winged helical domain (WHD) from the NOD module. The intramolecular interaction indicates that CNL ZAR1 in *Arabidopsis* adopts an inactive conformation (Wang et al., 2019a). In addition, ZAR1^LRR^ further inhibits ZAR1 by sequestering the NLR in a monomeric state, although the LRR-mediated inhibition is not essential for autoinhibition of some NLRs (Steele et al., 2019; Wang and Chai, 2020). A pair of Arabidopsis TNL proteins, RPS4 and RRS1, was shown to be required for recognition of AvrRps4 from the bacterial pathogen *Pseudomonas syringae* and PopP2 from *Ralstonia solanacearum*. RPS4 TIR domain forms homo-dimer and signals an effector-independent cell death. Co-expression of the RRS1 TIR domain, a RRS1/RPS4 TIR hetero-dimer competes with the formation of the RPS4 TIR homo-dimer to maintain the paired NLRs in an inactive state (Narusaka et al., 2009; Cesari et al., 2014a). Striking similarities are apparent between the RPS4/RRS1 and the RGA4 and RGA5 from rice functional models (Cesari et al., 2013; Cesari et al., 2014a; Cesari et al., 2014b).

After AVR recognition, the co-regulation of both NLR proteins exerts synergistic induction of immune responses for disease resistance (Eitas and Dangl, 2010; Okuyama et al., 2011; Zhai et al., 2014; Nishimura et al., 2015). The effector-triggered assembly of the CNL ZAR1 into pentameric resistosomes results in the formation of a calcium-permeable ion channel that integrates into the plasma membrane and initiates immune responses (Wang et al., 2019a; Wang et al., 2019b; Bi et al., 2021). Activated TNR and TNL use nicotinamide adenine dinucleotide (NAD^+^) or NAD^+^ with ATP as substrates to produce ADP-ribosylated adenosine triphosphate (ADPr-ATP) and ADPr-ADPR (di-ADPR) through ADP-ribosylation reactions, which are likely to be further hydrolyzed to pRib-ADP and pRib-AMP (2′-(5′′-phosphoribosyl)-5′-adenosine diphosphate and monophosphate). However, TNLs converge on the enhanced disease susceptibility 1 (EDS1) family of lipase-like proteins, which plays a key link between TNL activation and resistance pathway induction (Lapin et al., 2019). EDS1 forms heterodimers with phytoalexin deficient 4 (AtPAD4) and uses the same surface to interact with PAD4-related senescence associated gene 101 (SAG101). Together with the N requirement gene 1 (NRG1), a coevolved EDS1-SAG101-NRG1 module mediates cell death signaling by TIR-domain immune receptors (Lapin et al., 2019). The cryo-electron microscopy (cryo-EM) structure of TNL-activated EDS1-SAG101 revealed that TIR-catalyzed bioactive compounds, ADPr-ATP/ADPR and pRib-ADP/AMP, bind specifically between EDS1-PAD4 and EDS1-SAG101 dimers. The triggered conformational changes in PAD4 and SAG101 EP domains allosterically induce interactions with helper NLR NRG1A for plant resistance and cell death, respectively (Jia et al., 2022).

Paired NLR proteins exist widely in crops, such as *Oryza sativa*, *Zea mays*, *Triticum aestivum*, *Hordeum vulgare*, *Aegilops tauschii*, *Triticum urartu*, *Brachypodium distachyon*, and *Setaria italica*. In addition to having an intact CC/TIR-NB-ARC-LRR domain, they possess certain non-canonical IDs (Nishimura et al., 2015; Bailey et al., 2018; Stein et al., 2018). Most IDs are fused to the N- or C-terminus of NLR proteins (Cesari et al., 2013; Nishimura et al., 2015; Sarris et al., 2015). However, IDs are also present at both termini in certain NLR proteins (Ellis, 2016), or are located between the CC and NB domains in rare cases, such as the Pik-1 R protein of rice (Maqbool et al., 2015). NLR proteins carrying IDs can recognize pathogen effector proteins, known as sensor NLR (sNLR) proteins, and the protein partner that participates in the activation of downstream immune signaling is known as the helper NLR (hNLR) protein (Cesari et al., 2013; Maqbool et al., 2015; Sarris et al., 2015). Paired NLR proteins recognize pathogen effectors through direct physical contact with IDs or indirect interactions with the hNLR protein. Direct and indirect interactions between NLR proteins and pathogen effectors have been explained by various models, such as the receptor-ligand model (Keen, 1990), guard model (van der Biezen and Jones, 1998; Dangl and Jones, 2001; van der Hoorn and Kamoun, 2008), decoy model (Collier and Moffett, 2009; Dodds and Rathjen, 2010), and bait model (Collier and Moffett, 2009).

## 4 Structural basis of interactions between paired NLR-HMA IDs of rice and MAX-effectors of *M. oryzae*

Rice and *M. oryzae* constitute one of the model systems for studying plant–pathogen interactions. Currently, more than 100 *R* genes conferring resistance to blast disease have been mapped to the rice genome, of which more than 20 have been cloned. Except for the *R* genes *Pi-d2* and *pi21*, all the other genes encode NLR proteins (Liu and Wang, 2016). Interactions between the rice *R* genes and avirulence genes of *M. oryzae*, such as *Pib*/*AvrPib*, *Pi-ta*/*AvrPi-ta*, *Pi9*/*AvrPi9*, and *Piz-t/*A*vrPiz-t*, are consistent with the canonical gene-for-gene hypothesis (Flor, 1971). Researchers have also found that *R* gene pairs in rice, such as *Pik-1*/*Pik-2*, *RGA4*/*RGA5*, *Pi5-1*/*Pi5-2*, and *Pii-1*/*Pii-2*, jointly participate in defense responses (Lee et al., 2009; Yuan et al., 2011; Zhai et al., 2011; Cesari et al., 2013; Cesari et al., 2014a; Cesari et al., 2014b; Zhai et al., 2014; Maqbool et al., 2015; Takagi et al., 2017). Particularly, RGA5 and Pik-1, the respective sNLR proteins of *RGA4*/*RGA5* and *Pik-1*/*Pik-2*, depend on the conserved HMA IDs for the specific direct binding of effector proteins secreted by *M. oryzae*, thereby regulating hNLR-protein-triggered immune defense responses (Cesari et al., 2013; Zhai et al., 2014). HMA IDs are homologous to heavy metal-associated plant proteins and isoprenylated plant proteins (HIPPs). The conserved α/β sandwich structure of these proteins is comprised of two α-helices and four antiparallel β-sheets (topology: β1-αA-β2-β3-αB-β4), and heavy metal binding occurs at the conserved Cys-XX-Cys motif (Robinson and Winge, 2010; de Abreu-Neto et al., 2013). HMA IDs exist not only in the RGA4/RGA5, Pik-1/Pik-2, and pi21 R proteins of rice and participate in immune responses, but also in the NLR receptor proteins of flowering crops belonging to the Brassicaceae, Fabaceae, and Rosaceae families (Kroj et al., 2016; Sarris et al., 2016). They have also been identified in other plant proteins that are unrelated to NLR proteins (de Abreu-Neto et al., 2013).

Accumulating evidence demonstrated that the relative location and sequence diversity of HMA IDs in the RGA4/RGA5 and Pik-1/Pik-2 proteins of rice exert important effects on their biological functions (**Table 1**) (Cesari et al., 2013; Zhai et al., 2014; Maqbool et al., 2015). HMA IDs are located separately downstream of the RGA5 LRR domain (RGA5-HMA), but between the CC and NB-ARC domains at the N-terminus of Pik proteins (Pik-HMA), they showed sequence similarities of up to 54% (Cesari et al., 2013; Maqbool et al., 2015; Guo et al., 2018). The hNLR RGA4 acts as an auto-active inducer of immune signaling (Cesari et al., 2014b). RGA4 and RGA5 form homo- and hetero-complexes and interact through their CC domains. While the RGA5 CC domain is necessary but not sufficient for repression of RGA4-mediated cell death, its HMA IDs is dispensable (Cesari et al., 2013; Cesari et al., 2014a; Cesari et al., 2014b). RGA5-HMA IDs can simultaneously recognize the effectors AVR1-CO39 and AVR-Pia through directly binding, which is repressed by RGA5 in the absence of effectors (Cesari et al., 2014b). Upon *M. oryzae* invasion, the binding of AVR1-CO39 and AVR-Pia to the self-interacting αα/β2 interfaces of RGA5-HMA IDs competes with RGA5-HMA self-interaction, which forms a heterocomplex with 1:1 stoichiometry and relieves RGA4 repression (Guo et al., 2018). The β2 of RGA5-HMA IDs is aligned in an antiparallel manner with the β2 of AVR1-CO39, forming a continuous antiparallel β-sheet comprising the four-stranded β-sheet of RGA5-HMA IDs and β1, 2, and 6 of AVR1-CO39 (Guo et al., 2018). However, in AVR-Pia, the HMA-binding interface is more extended. In addition to the β2 of RGA5-HMA IDs, both β3 and the loop formed between β2 and β3 also participate in interactions during binding to AVR-Pia (Guo et al., 2018).

**Table 1:** HMA IDs of rice NLR protein pairs and the corresponding MAX-effectors of *M. oryzae*.

NLR pairs	sNLR	HMA IDs location	MAX-effectors	Binding mode	References
RGA4/RGA5	RGA5	C-terminus of RGA5	AVR1-CO39 AVR-Pia	1:1	Cesari et al., 2013; Guo et al., 2018
Pikp-1/Pikp-2	Pikp-1	between CC and NB-ARC	AVR-PikD	2:1	Kanzaki et al., 2012; Maqbool et al., 2015; De la Concepcion et al., 2018, 2021; Xiao et al., 2022
Pikm-1/Pikm-2	Pikm-1		AVR-PikD/E/A	1:1	
Piks-1/Piks-2	Piks-1		AVR-PikD	unknown	
Pikh-1/Pikh-2	Pikh-1		AVR-PikD/E/A	2:1	

HMA IDs; heavy metal-associated (HMA) integrated domains (IDs). CC; coiled-coil. NB-ARC; nucleotide-binding adaptor shared by the human apoptotic regulator APAF-1, plant resistance (R) proteins and Caenorhabditis elegans CED-4.

Co-evolutionary dynamics have driven the emergence of five different Pik alleles, Pikh, Pikp, Pikm, Piks, and Pik*, to display the extended recognition of AVR-Pik (A to F) variants (Yoshida et al., 2009; Kanzaki et al., 2012; Zhai et al., 2014; De la Concepcion et al., 2021; Xiao et al., 2022). AVR1-CO39, AVR-Pia, and AVR-Pik variants have no apparent sequence identity but are highly similar to the MAX (*Magnaporthe* AVRs and ToxB-like) effector family, which adopts a six β-sandwich fold stabilized by buried hydrophobic residues and commonly, but not always, disulfide bonds (de Guillen et al., 2015; Zhang et al., 2018). Although RGA5 and Pik-1 both display canonical HMA IDs formed in dimer states, structure-function analyses involving nuclear magnetic resonance and x-ray crystallography reveal that the intramolecular interaction mechanisms and binding interfaces are completely different in the two HMA IDs/MAX-effector systems (Maqbool et al., 2015; Cesari, 2018; De la Concepcion et al., 2018; Guo et al., 2018).

**Figure 1:**
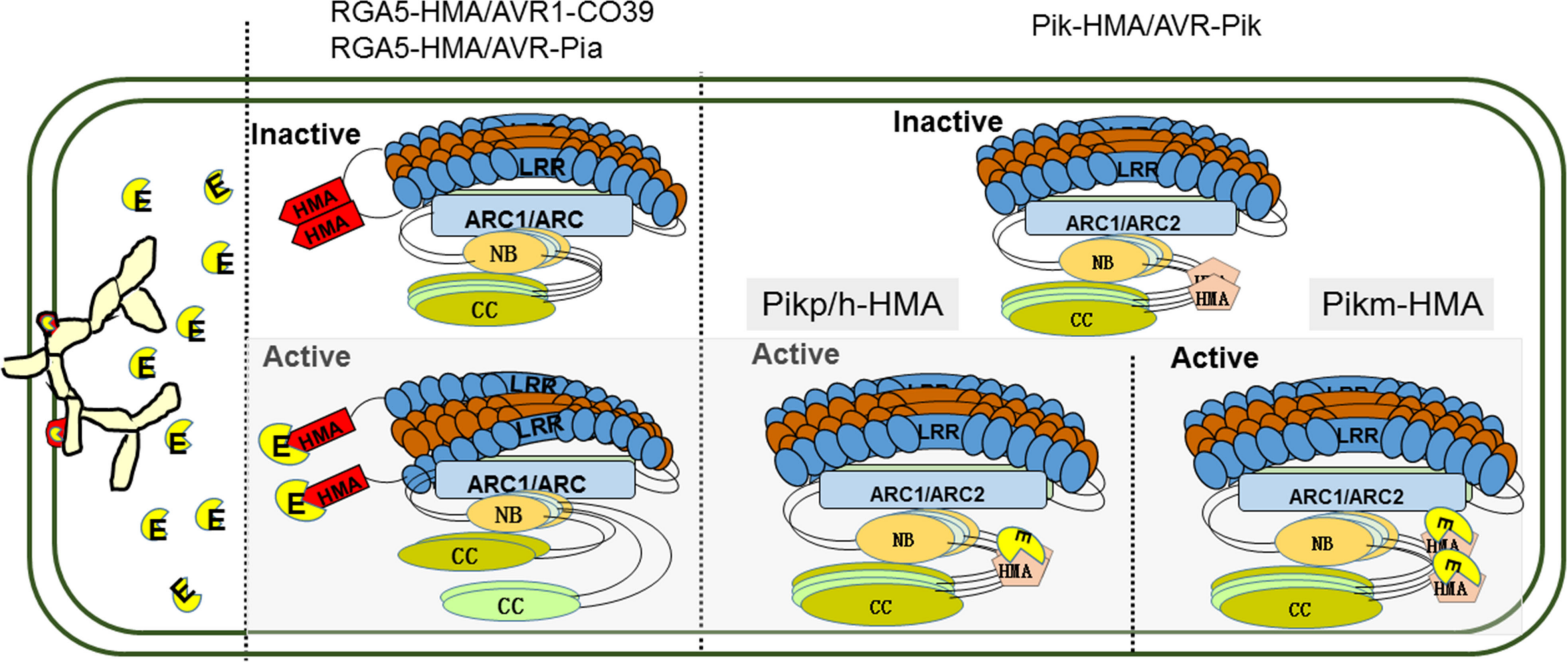
Binding modes for the diverse recognition of paired NLR-HMA IDs/MAX-effectors. In the absence of pathogens, NLR paired proteins are kept in an inactive state by forming a hetero-complex, and the HMA IDs forms a homodimers. After pathogen infected, the secreted effector proteins directely bind to sNLR like molecular traps and activate the immune. For RGA4/RGA5 (blue and brown), the effectors of AVR1-CO39 and AVR-Pia (yellow) binding to RGA5-HMA IDs (red) competes with RGA5-HMA self-interaction, which forms a heterocomplex with a 1:1 stoichiometry and relieves RGA4 repression. Like the RGA5-HMA/effector binding systems, AVR-PikD/E/A compete with Pikm-HMA self-interaction in a 1:1 binding mode; but directly bind to Pikp/h-HMA forming a 2:1 complex. E; effectors secreted by *Magnaporthe oryzae*. HMA IDs; heavy metal-associated (HMA) integrated domains (IDs). NLR; nucleotide-binding and leucine-rich repeat. MAX-effectors; *Magnaporthe oryzae* AVRs and ToxB-like.

In contrast to RGA4, expression of Pik-2 dose not constitutively activate cell death. HMA IDs in sNLR Pik-1 requires the hNLR Pik-2 to initiate signaling, suggesting that the assembly of an active signaling complex requires all three proteins (Kanzaki et al., 2012; Maqbool et al., 2015). Structure-informed similarity searches showed that the co-evolution between pathogens and hosts has driven the emergence of two different binding modes among the Pik-HMA/AVR-Pik systems through multiple interfaces (**Figure 1**) (Maqbool et al., 2015; De la Concepcion et al., 2018; De la Concepcion et al., 2019; Xi et al., 2022). (1) Like the RGA5-HMA/effector binding systems, AVR-PikD/E/A competes with Pikm-HMA self-interaction in a 1:1 binding mode (De la Concepcion et al., 2019). (2) Pikp-HMA/AVR-PikD/E and AVR-Pikh/AVR-PikC form a 2:1 complex, but do not compete with Pikp/h-HMA self-interaction (**Figure 1**). The figure is optimized basing the functional models of RGA4/RGA5 NLR pair (Cesari et al., 2014a). Further analysis showed that the overall orientations of each component in the Pikm-HMA/effector complexes are similar to each other, and to determine Pikp-HMA/AVR-PikD/E and Pikh-HMA/AVR-PikC. However, the core residues contributing to the interaction are significantly different. Within the Pikp-HMA/AVR-PikD complex, Pikp-HMA^Ser218,Gly230^ binds to AVR-PikD^His46^ via hydrogen bonds, forming the core region for the regulation of their interactions. In the Pikm-HMA/AVR-PikD complex, G230 is replaced by Val231 at a structurally equivalent position in the Pikp-HMA structure. Therefore, Pikm-HMA^Val231^ cannot directly form salt bridges with AVR-PikD^His46^. In the Pikm-HMA/AVR-PikE complex, the amino acid at position 46 of the effector protein is asparagine (Asn). Consequently, Asn is rotated out of the binding pocket and is located well away from Pikm^Val231^, and hydrogen bonds are formed between AVR-PikE^(Asn46:N^<σπ>δ</σπ>^2)^ and both Pikm-HMA^(Ser219:OH)^ and the new water molecule. Such a configuration affects the position of AVR-Pikm^Phe44-Gly48^ and pushes them away from Pikm-HMA IDs, further altering the interactions across the core interface. Such structural changes are related to the decreased binding affinity of AVR-PikE to Pikm-HMA compared with that of AVR-PikD (De la Concepcion et al., 2018). In the Pikm-HMA/AVR-PikA coordination complex, Asn46 is rotated further out of the HMA pocket. Although a hydrogen bond is still formed with Pikm-HMA^(Ser219:OH)^, a considerable difference occurs in orientation. These changes cause the residues of AVR-PikA^Asn46 - Pro50^ to be located a distance from Pikm-HMA, and such structural observations are again related to decreased effector binding affinity (De la Concepcion et al., 2018). The research results described above indicate that sequence diversity is a key factor affecting the interactions between MAX-effectors of *M. oryzae* and paired NLR-HMA IDs in rice and the recognition of MAX-effectors, thereby affecting biological functions of paired NLR-HMA IDs.

## 5 Applications of paired NLR-HMA IDs in rice designed based on their structure

Engineering made-to-order plant immune receptors has been confirmed as a potential method to confer plant disease resistance (Kourelis et al., 2023). Targeted point mutations or random mutational screens have succeeded in extending NLR recognition specificity or increasing their activation properties to create sensitized NLR (Harris et al., 2013; Segretin et al., 2014). Therefore, the use of molecular engineering to design *R* genes for the creation of new specificities or expansion of NLR protein recognition profiles has great potential in crop genetics and breeding (Kim et al., 2016; Cesari, 2018; Grund et al., 2019; Kourelis et al., 2021; Cesari et al., 2022). A classic example is the alternative strategy for modifying the protein kinase PBS1 in *Arabidopsis thaliana* (Kim et al., 2016). It is believed that PBS1 acts as a bait in the system, similar to the mousetrap model. The NLR protein RPS5 (resistance to *Pseudomonas syringae*) forms a pre-activation complex with PBS1. The cleavage of PBS1 by AvrPphB (Gly-Asp-Lys-Ser-His-Val-Ser) secreted by the pathogenic oomycete *Phytophthora infestans* leads to conformational changes in PBS1, which serve as a key factor in triggering RPS5-induced HR (DeYoung et al., 2012). Thus, replacement of the cleavage site of PBS1 with that of protease effector proteins of other bacteria and viruses (PBS^RCS2^ and PBS^TuMV^) can also trigger RPS5 activity, thereby expanding the recognition specificity of the host towards pathogens (Kim et al., 2016; Pottinger et al., 2020).

The use of structural biology to elucidate the molecular basis of the interactions between plant NLR proteins and pathogen effector proteins at the atomic level provides new approaches to achieve precise NLR protein design through targeted mutations. The highlights of the potential of HMA-IDs engineering in rice are as follows: (1) in the RGA4/RGA5 and Pik-1/Pik2 systems, HMA IDs can act as sNLR, binding MAX-effectors like molecular traps and activating the immune response of the system; (2) RGA5-HMA IDs and Pik-HMA IDs show 54% sequence similarity; (3) structure-function analysis demonstrated high plasticity in HMA ID and MAX-effectors interactions. Because their sequence is unrelated but structurally similar, MAX-effectors bind to different surfaces of structurally conserved HMA IDs. Therefore, the design of HMA IDs with expanded recognition profiles based on HMA IDs/MAX-effector complex structures has immense potential and has achieved promising progress (**Table 2**).

**Table 2:** Structure-informed engineering of paired NLR-HMA IDs in rice.

Wild-type		Engineering of HMA IDs		References
HMA-ID	Corresponding MAX-effectors	HMA-IDs mutation	Corresponding MAX-effectors	
Pikp-HMA	AVR-PikD	Pikp-HMA^Asn261Lys, Lys262Glu^	AVR-PikD/E/A	De la Concepcion et al., 2019
Pikm-1	AVR-PikD AVR-PikE AVR-PikA	Pikobody	*Potato virus X (*PVX)	Kourelis et al., 2023
RGA5-HMA	AVR1-CO39, AVR-Pia	RGA5-HMA^Sel1027Val, Gly1009Asp,^ (RGA5-HMA2)	AVR-Pib	Liu et al., 2021
RGA5-HMA ^(E1029A, I1030L, T1031V, E1033D, D1034L, K1035R, R1037K, L1038I, V1039E)^ (RGA5-HMAm1)		AVR1-CO39 AVR-Pia AVR-PikD	Cesari et al., 2022
RGA5-HMA ^(M1065Q, E1067S, L1068Q)^(RGA5-HMAm2) RGA5-HMA ^(E1029A, I1030L, T1031V, E1033D, D1034L, K1035R, R1037K, L1038I, V1039E, M1065Q, E1067S, L1068Q)^(RGA5-HMAm1m2)		

NLR; nucleotide-binding and leucine-rich repeat. HMA IDs; heavy metal-associated (HMA) integrated domains (IDs). MAX-effectors; Magnaporthe oryzae AVRs and ToxB-like. m: mutation.

De la Concepcion et al. (2019) analyzed and compared the key residues that participate in AVR-Pik interactions in the crystal structures of the complexes. By constructing key mutations in the interface 2 and 3 regions of Pik-HMA, or swapping residues found in Pikm into Pikp, the researchers obtained the mutant Pikp^Asn261Lys,Lys262Glu^, with an expanded AVR-Pik recognition profile for the first time. Besides having enhanced AVR-PikD recognition ability, the mutation could also recognize AVR-PikE and AVR-PikA. Maidment et al. (2021) demonstrated that the OsHIPP19-HMA protein in rice, which is related to cold/drought tolerance and possesses conserved domains, can bind to multiple effector proteins (AVR-PikD, C, F) in a similar HMA IDs/MAX-effector mode. This study provides an important molecular basis for designing Pik-1 receptor mutants similar to OsHIPP19-HMA IDs, with an expanded Pik resistance profile. RGA5-HMA-ID modifications have also received considerable research attention, with researchers successfully creating the RGA5-HMA^Sel1027Val,Gly1009Asp^ (RGA5-HMA2) mutant with new AVR-Pib recognition sites (Liu et al., 2021) and obtaining RGA5-HMA^(E1029A,I1030L,T1031V,E1033D,D1034L,K1035R,R1037K,L1038I,V1039E)^ (RGA5-HMAm1) and RGA5-HMA^(E1029A,I1030L,T1031V,E1033D,D1034L,K1035R,R1037K,L1038I,V1039E,M1065Q,E1067S,L1068Q)^ (RGA5-HMAm1m2) with expanded MAX-effector recognition profiles (Cesari et al., 2022). Moreover, the HMA-IDs of Pikm-1 have been successfully swapped with nanobody (single-domain antibody fragment) fusions that bind either green fluorescent protein (GFP) or mCherry (Kourelis et al., 2023). Pikobody is shortened for Pikm-1-nanobody fusions with GFP or mCherry. The bioengineered Pikobody confers new-to-nature resistance activities against *Potato virus X (*PVX) expressing GFP or mCherry (Kourelis et al., 2023). These results demonstrate that the HMA-IDs-based design of rice materials with broad resistance profiles possesses great application potential but also faces considerable challenges.

For the structure-guided engineering of immune receptors, *R* gene stacking deserves concern. Functional stacking of resistance genes has been used in plant breeding strategy to increase durability and confer broad-spectrum resistance (Ghislain et al., 2019; Luo et al., 2021; Kourelis et al., 2023). Stacking of three late blight *R* genes in potato varieties confers extreme resistance to late blight (Ghislain et al., 2019). A five-transgene cassette has been successfully introduced into bread wheat and shows very high level of resistance to a fungal rust pathogen in wheat (Luo et al., 2021). However, overaccumulation of NLRs often leads to autoimmune responses, suggesting that the levels of these immune receptors must be tightly controlled (Cheng et al., 2011). In addition, gain-of-function mutants of NLRs, constitutive expression of defense marker *Pathogenesis Related* (*PR*) genes, enhanced pathogen resistance, and altered plant development, such as dwarfism, are general features of plant autoimmunity (Cheng et al., 2011). Therefore, NLRs should be maintained at proper levels to prevent autoimmunity (Cheng et al., 2011). Besides, in some hybrid plants, independently evolved immune system components are mismatched and trigger inappropriate immune reactions in the absence of pathogens (Tran et al., 2017). NLR misactivation has been linked to autoimmunity and induces a typical hybrid necrosis (Chakraborty et al., 2018).

## 6 Conclusions and prospects

Coordinated development of structural biology and genetics has provided an important molecular basis for elucidating the interactions between plant NLR proteins and pathogens. In this review article, we summarize the importance of the plant immune system and the progress in genetic engineering strategies for the targeted design of HMA IDs with expanded, enhanced, or new MAX-effector recognition abilities based on the structural information of paired NLR-HMA IDs in rice and MAX-effectors of *M. oryzae*. Therefore, adequate use of the immune systems of crops and in-depth exploration of the interaction modes of NLR proteins and effectors provide an important molecular basis for the mining and designing of *R* genes and resistant crop varieties. However, the following issues require further consideration and investigation:

- Transgenic rice varieties conferring increased or extended anti-rice blast specificity by modified HMA IDs are yet to be developed.
- Exploration of the feasibility of designing HMA IDs through modification or exchange to confer recognition profiles for MAX-effectors of other pathogens besides *M. oryzae*. It is unclear whether continuous resistance can be provided by transplanting engineered HMA IDs into crops belonging to related or unrelated genera of rice.
- Elucidation of the molecular mechanisms of synergistic interactions in the paired NLR proteins of rice to determine how paired NLR-HMA IDs activate sNLR proteins to induce molecular transduction of hNLR immune signals.
- Exploration of the molecular mechanisms and modification strategies for HMA IDs/MAX-effectors as models for further application to other crops for in-depth investigation of the diversity of NLR proteins in recognizing effectors and activating immune mechanisms.

## Author contributions

XH and JL conceived and designed the review. LG and YM wrote the manuscript. Other authors listed have made a substantial direct and intellectual contribution to the work and approved it for publication.
